# The Application of Next-Generation Sequencing to Define Factors Related to Oral Cancer and Discover Novel Biomarkers

**DOI:** 10.3390/life10100228

**Published:** 2020-10-02

**Authors:** Soyeon Kim, Joo Won Lee, Young-Seok Park

**Affiliations:** Department of Oral Anatomy and Dental Research Institute, School of Dentistry, Seoul National University, Seoul 03968, Korea; babyyoda@snu.ac.kr (S.K.); europa@snu.ac.kr (J.W.L.)

**Keywords:** OSCC, next-generation sequencing, biomarker, targeted therapy

## Abstract

Despite the introduction of next-generation sequencing in the realm of DNA sequencing technology, it is not often used in the investigation of oral squamous cell carcinoma (OSCC). Oral cancer is one of the most frequently occurring malignancies in some parts of the world and has a high mortality rate. Patients with this malignancy are likely to have a poor prognosis and may suffer from severe facial deformity or mastication problems even after successful treatment. Therefore, a thorough understanding of this malignancy is essential to prevent and treat it. This review sought to highlight the contributions of next-generation sequencing (NGS) in unveiling the genetic alterations and differential expressions of miRNAs involved in OSCC progression. By applying an appropriate eligibility criterion, we selected relevant studies for review. Frequently identified mutations in genes such as *TP53*, *NOTCH1*, and *PIK3CA* are discussed. The findings of existing miRNAs (e.g., miR-21) as well as novel discoveries pertaining to OSCC are also covered. Lastly, we briefly mention the latest findings in targeted gene therapy and the potential use of miRNAs as biomarkers. Our goal is to encourage researchers to further adopt NGS in their studies and give an overview of the latest findings of OSCC treatment.

## 1. Introduction

### 1.1. Next-Generation Sequencing

Next-generation sequencing (NGS) is a powerful tool that has enabled the systematic study of genomes and provided researchers with insights into disease understanding. One major advantage of NGS is the ability to sequence the entire genome or targeted areas in a short amount of time [1]. Sanger sequencing, which was developed by Frederick Sanger in the 1970s, was the first commercialized sequencing method that eventually gave rise to the revolutionary NGS method. Sanger developed the “plus and minus” sequencing method, which involved the use of bacteriophage phiX174, and the “DNA sequencing with chain-terminating inhibitors” [2,3]. The Maxam–Gilbert sequencing method was another conventional method that preceded NGS. This method uses radioactive labeling at the 5′ end of the DNA, and does not require cloning of the DNA strand before the sequencing [4]. However, the Maxam–Gilbert method is no longer widely adopted due to its use of hazardous chemicals. Though Sanger sequencing is still used today, the preference for NGS is rapidly increasing because of its overall performance and the many advantages it offers compared to conventional sequencing methods.

Among the various NGS platforms, the most preferred platforms are Illumina/Solexa and Ion Torrent. The low cost, high yield, and wide availability of Illumina/Solexa are the main reasons for their high demand [5,6]. The Ion Torrent, which is another highly sought after NGS method, has short run times and longer read lengths than the Illumina [6]. Despite the emergence of Roche 454 and ABI/SOLiD in the late 2000s, they are no longer supported due to price concerns and short read lengths, respectively [5,6]. Two relatively new platforms, Oxford Nanopore Technologies (ONT) and Pacific Biosciences (PacBio), have been developed to read longer lengths [7]. These are capable of sequencing the entire cDNA in a single run [8]. Recently, Bejing Genomics Institute (BGI)’s BGISEQ was introduced to the market. Using a DNA nanoball technology, this platform generates up to a few terabases of sequenced data in a single run [9,10,11]. The low cost and large-scale DNA sequencing ability are comparable to Illumina, and researchers have sought to compare the performances of BGISEQ and Illumina [10,12].

Various fields have benefitted greatly from the advent of NGS. In microbiological studies, NGS has substantially improved the genomic approach of pathogen investigation. The oral microbiota is the second largest microbial community found in the human body. Therefore, identifying the types of microorganisms that inhabit the oral cavity is essential for maintaining good health. The following two NGS approaches have been used to evaluate the microbiota of the oral cavity: 16S rRNA sequencing and shotgun sequencing [13]. Since the 16S rRNA gene is present in all bacteria, it can be used to determine the presence of bacteria in the oral cavity and differentiate them based on variations across species [14]. Shotgun sequencing is performed on the whole microbial genome and reveals additional information, such as the metabolic pathways of each species and the resistance/virulence genes [15]. The ability to classify the entire microbial genome, instead of targeting specific taxa, is a major progress in genomics. Thus, the NGS approach has shed light upon the genomic underpinnings of microbes and drug development.

In addition to the extensive use of NGS in microbiological studies, it has elucidated the ambiguities of cancer genomics. Researchers have relied on Sanger sequencing or PCR-based assays in the past, both of which have limitations compared to NGS. The Sanger method has a smaller processing volume size and lower sensitivity than NGS. The minimum mutant allele frequency for Sanger sequencing is around 20% to 25%, although mutations below the minimum could be detected depending on the targeted sequence [16,17]. Real-time quantitative PCR (qRT-PCR), despite its high sensitivity and speed, can only detect a few known sequences [18]. Unlike other sequencing approaches, NGS allows for whole exome sequencing of tumors, targeted sequencing (revealing key genes associated with cancer), and RNA sequencing [19]. Another important feature of NGS is its role in dealing with tumor heterogeneity [20,21]. Most solid tumors have tumor heterogeneity, making diagnosis and treatment extremely challenging [22]. Oral squamous cell carcinoma (OSCC) is a type of solid tumor characterized by tumor heterogeneity and thus requires substantial research at the genomic level [20,21]. The NGS method in this regard is essential for detecting mutations with low variant allele frequency.

Through NGS, various known and previously unknown genetic alterations have been identified. As the application of NGS is shifting from research settings to clinical ones, this review discusses the recent findings of NGS-applied OSCC research and drug development for OSCC. We also discuss several of the leading causes of OSCC, including gene alteration and the differential expression of miRNAs, which may serve as putative biomarkers.

### 1.2. Oral Squamous Cell Carcinoma

OSCC is somewhere between the sixth to eighth most commonly occurring malignancy in the world, and the incidence is estimated to be an average of 4 cases per 100,000 people [23,24,25,26]. South Asian countries, such as Pakistan, Bangladesh, India, and Sri Lanka, are at further risk mainly due to tobacco, alcohol, and betel quid use [23]. In these countries, one-third of all reported cancers are oral cancer [27]. In Western countries, the leading risk factors (i.e., tobacco, alcohol, and smoking), as well as human papilloma virus (HPV), are attributed to an increase in the number of OSCC patients among the younger population [23].

Oral cancer can develop in the palate, inside of the cheeks, lips, tongue, or gums. Classified by the type of cell that proliferates, most oral malignancies are classified as squamous cell carcinoma. The oral squamous cell carcinoma (OSCC) accounts for more than 90% of all tumorigenesis in the oral cavity [28,29]. OSCC tends to occur more frequently in men than in women, and it usually occurs after age 50 [30].

The most common precancerous OSCC lesions are leukoplakia, erythroplakia, and oral submucous fibrosis (OSMF) [31,32,33]. Leukoplakia appears as a white patch or plaque, and tobacco chewing is known as the main risk factor leading to this condition [32,33]. Although the transformation rate of leukoplakia to OSCC varies, a study comparing the premalignant lesions of OSCC showed that leukoplakia was the precursor in 20% of the cases, while the others were negligible (less than 1%) [34]. Identifying the precursors of OSCC is crucial to maximize treatment results, as the failure to detect premalignant lesions reduces the five-year survival rate down to 50% [35,36].

As with most malignancies, metastasis is inevitable if OSCC is identified at a late stage. There is a high risk of cervical lymph node metastasis even when the primary tumors are small [37]. The recurrence rate of OSCC is quite high, and lymph node metastasis occurs in about 40% of OSCC patients [38]. Cervical metastasis determines the prognosis of OSCC. With metastasis, the five-year survival is reduced from 90% to 20–25% [39,40].

Besides the evident dangers of OSCC, some interesting trends for this malignancy should be addressed. The statistics and research outcomes have shown an increasing incidence of oral cancer in younger patients, while the overall incidence of OSCC has been decreasing [41,42,43,44,45,46,47]. The increase in younger OSCC patients alone is problematic, and the fact that the known risk factors for oral cancer do not necessarily explain this phenomenon is even more concerning. Furthermore, alterations in particular genes tend to be more prevalent in certain Asian populations with OSCC than they are in other ethnic groups, although further research is needed to confirm these findings [48,49].

## 2. Gene Alterations

In recent years, researchers have embraced the technology of NGS and discovered numerous genes that can lead to OSCC when they are mutated (Table 1). Previous studies have revealed that *TP53*, *CDKN2A*, *NOTCH1*, *FBXW7, HRAS*, and *PIK3CA* genes were frequently mutated in patients with head and neck squamous cell carcinoma (HNSCC) [50,51].

**Figure 1:**
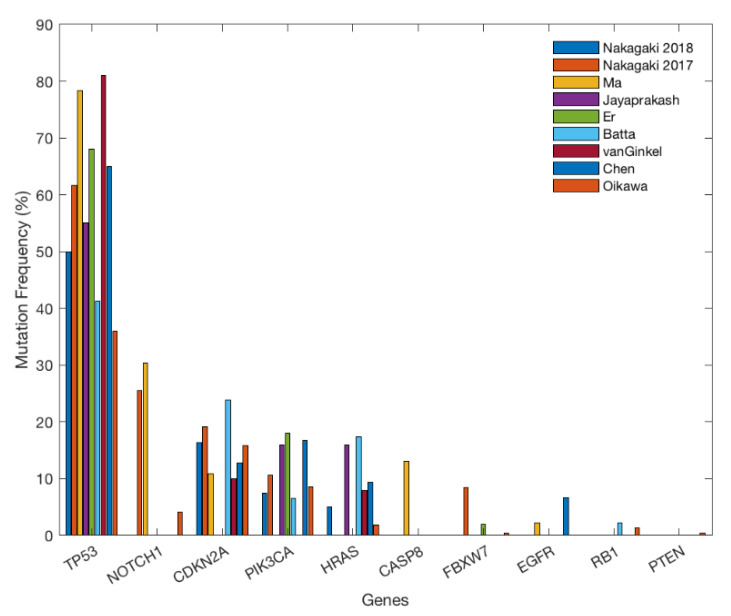
The percentage of affected samples in each gene. *TP53* mutations are the most prominent driver mutations of OSCC, followed by *NOTCH1*, *CDKN2A*, *PIK3CA*, *HRAS, CASP8*, and *FBXW7*. *EGFR, RB1*, and *PTEN* mutations were also identified by several studies, but the mutation frequencies of each gene in individual studies were not high.

As reported by many oncological studies, mutations in the *TP53* gene are present in more than half of human cancers [52]. *TP53* is also the most frequently mutated gene in OSCC, with frequencies ranging from 35.9% to 81% [48,53,54,55,56,57,58,59,60,61,62] (Figure 1). The high mutation rate is significant because *TP53* mutation is believed to initiate the proliferation of mutated cells [62]. Among the various mutations observed in *TP53*, missense mutations are the most common [48,53,54,55,56,57,58,59,60,61,63]. Furthermore, most *TP53* mutations are located near the DNA binding domain of the protein, which is highly associated with poor prognosis [53,54,56]. Some studies investigated the relationship between gene mutations and HPV [53,59,64]. Although there is no distinct relationship between p53 and HPV status, *TP53* mutations occurred more frequently in HPV negative OSCCs than they did in HPV positive OSCCs [53,64]. In addition, mutant *TP53* was rarely found in OSCC specimens that contained HPV16 DNA [53]. In one study, *TP53* mutation was only observed in an HPV negative subgroup in HNSCCs [65].

The *PIK3CA* gene is another commonly mutated gene in OSCC [53,54,55,58,59,60,61,63,66]. The frequency ranges from 10% to 23.9%, which is less than the mutation frequency of *TP53* [53,54,55,57,58,60,63]. Unlike *TP53*, the *PIK3CA* gene mutations are commonly observed in HPV positive oral cancers [65,67]. Studies also suggested that the most common mutational hotspots of *PIK3CA* are located in exon 20 [58,63,66], and a regional association between the *PIK3CA* mutation and the tumor site (i.e., lower alveolus and lower lip) has been observed [58]. According to some studies, *PIK3CA* mutations may be associated with the later stages of OSCC, as *PIK3CA* is frequently mutated in stage IV OSCC [54,66]. Evidence suggests that the RTK/MAPK/PI3K pathway is a putative target for treating OSCC, because changes to this pathway result in poor survival [54]. Similarly, the deregulation of PI3KCA (due to mutation) leads to activation of the PI3K/Akt signaling pathway. These findings suggest that targeting the PI3K/Akt signaling pathway might improve the diagnosis and treatment of OSCC [55]. Altogether, the heterogeneous findings regarding *PIK3CA* mutations provide an opportunity for the development of various therapies and treatments.

Known for its tumor-suppressive role in OSCC, the *NOTCH1* gene may also promote OSCC initiation or progression when it is altered [48,49,53,54,56,59,60,63,68]. Of the studies included for review, most reported an approximately 22–30% mutation frequency [54,56,59] in *NOTCH1*, except one study that reported a 4.1% frequency [60]. One study discovered that *NOTCH1* was the second most frequently mutated gene in Japanese patients suffering from OSCC [54]. Interestingly, past studies have also shown a high frequency of *NOTCH1* mutations in other Asian populations with OSCC [48,49]. *NOTCH1* mutations occur more frequently in Asian patients (specifically Chinese) than they do in Caucasian patients. This cultural difference may be attributable to the types of carcinogens that the patients were exposed to (including possible higher alcohol intake among Chinese) [48]. However, cultural differences may not be the only factor, as slight variations of *NOTCH1* mutations have also been observed among different races. The majority of mutations identified in Asian patients were observed within the EGF-like repeats, especially near the ‘ligand-binding’ and Abruptex regions [49]. It is known that mutations in these regions hinder the function of *NOTCH1* [49]. In contrast, mutations in Caucasian patients were most prevalent in the ‘ligand-binding’ domains, which indicates that the cause for *NOTCH1*-related oral tumorigenesis was most likely the inhibition of the *NOTCH1–*ligand interaction [49]. These findings suggest that different mutation sites may be characteristic of particular races. Lastly, a mutation in *NOTCH1* was characterized by poor survival [48,63].

Despite the efforts to identify mutations responsible for OSCC, the challenges of treating this malignancy remain due to tumor heterogeneity. Studies have revealed that cancer genomes differed among specimens taken from different areas of the malignancy, suggesting intratumoral heterogeneity (ITH) [21,69]. Intratumoral heterogeneity refers to the heterogeneous nature of individual tumor cells (both morphological and genotypical differences) within the same tumor. This concept was first introduced by Slaughter and his colleagues [21,70]. They found that 88 (11.2%) of 783 patients exhibited at least two different morphological features of OSCC [71]. In addition, about half of the 88 patients had two separate tumors in the same anatomical region of tumor growth [71]. Based on this discovery, subsequent studies have revealed the existence of ITH through advanced genomic research methods [21,70]. One study performed NGS to confirm tumor heterogeneity in HNSCC patients and successfully demonstrated that the biopsy of a single tumor site may not be sufficient to understand the whole genomics of HNSCC [72]. Furthermore, some researchers sought to determine the relationship between intratumoral heterogeneity and field heterogeneity (FH) using NGS [21]. Their results suggest that FH might have greater impact on OSCC outcome than ITH [21]. Altogether, these findings implicate the importance of personalized medicine for OSCC and the consideration of both ITH and FH in these processes.

## 3. Targeted Gene Therapy

Numerous therapeutic drugs targeting specific genes, proteins, and enzymes related to OSCC are currently available (Table 2). The p53-targeting drugs include PRIMA-1, PRIMA-1^MET^(APR-246), MIRA-1, STIMA-1, and COTI-2. [73,74]. These compounds restore p53 to its wild-type conformation, thereby reactivating the transcriptional activity of wt-p53 [52,74]. The cysteine-binding PRIMA-1 and APR-246 induce apoptosis through caspase activation [75]. The efficacy of PRIMA-1 and APR-246 has been demonstrated in many studies investigating various malignancies, but only a few studies have practiced them on OSCC/HNSCC treatment so far [76,77]. Although the studies have confirmed the p53 restoring ability of PRIMA-1 and APR-246, experimental outcomes suggest that their anti-cancer characteristics might be independent of p53 restoration [78]. STIMA-1 and MIRA-1 are also cysteine-binding compounds that prevent unfolding of p53 (both wild-type and mutant forms) [79]. However, they have not been tested on HNSCC yet, and the performance of MIRA-1 was not as strong as PRIMA-1 [80,81]. The relatively new COTI-2 is a Zn^2+^ chelating compound that induces proper folding of p53 [79,82,83]. The efficacy of COTI-2 was demonstrated in numerous types of cancer, including OSCC [82,83]. It is currently under evaluation in a phase I clinical study of gynecological cancer and HNSCC [82,83]. In an in vitro study, COTI-2 was successful at inhibiting OSCC tumor growth [82]. Some researchers believe that COTI-2 is more effective than cetuximab and erlotinib in terms of anti-proliferative properties [84].

The drugs that target the epidermal growth factor receptor (*EGFR*) consist of two subgroups depending on their targeting mechanism. Cetuximab and nimotuzumab function as monoclonal antibodies (IgG1) against *EGFR* [88]. These medications regulate the stability of the *EGFR* protein through the ubiquitin/proteasome pathway, thereby reducing tumor cell proliferation and migration [89]. Cetuximab in conjunction with radiotherapy received FDA approval for treatment of HNSCC [85]. A retrospective study used cetuximab to treat OSCC and the effective rate for locally advanced and recurring OSCC was 57.1% [85]. In addition, the effective rate for patients with distant metastasis was 60% [85]. Nimotuzumab also demonstrated efficacy in treating OSCC when used in combination with chemoradiotherapy [85]. The *EGFR* tyrosine kinase inhibitors, gefitinib, erlotinib, and afatinib are currently under clinical trials for treating OSCC/HNSCC [85]. Gefitinib inhibits *EGFR* by increasing the apoptotic function of cisplatin [90]. Although it showed efficacy especially on metastatic/recurrent OSCC when used with paclitaxel and chemotherapy, problems with toxicity question its further application [85,91]. Afatinib and erlotinib prevent the growth of HNSCC cells by inhibiting *EGFR*1 phosphorylation [92]. According to a study, erlotinib may be effective in reducing precancerous lesions of OSCC, but not in severely progressed OSCC [93].

Vascular endothelial growth factor (VEGF) and its receptors are major contributors to angiogenesis, a process that significantly impacts tumor progression and metastasis [94]. High expressions of VEGF were observed in OSCC specimen studies, which suggests that VEGF is an important OSCC biomarker [94]. As a result of these findings, multiple anti-angiogenic drugs have been developed [94]. Some of the anti-angiogenic drugs or inhibitors of VEGF currently under investigation include bevacizumab, sorafenib, aflibercept, and vandetanib [85]. Bevacizumab injections decreased tumor growth in OSCC xenografts [95]. In a study comparing bevacizumab and aflibercept, the migration rate of cells was much lower when aflibercept was applied [87]. Sorafenib, used in combination with radiotherapy, suppressed NF-κB and associated proteins, which are involved in tumorigenesis and radioresistance [96]. Vandetanib inhibits both V*EGFR*-2 and *EGFR* tyrosine kinase [97,98]. This drug also demonstrated promising results in various studies, whether it was used independently or in combination with photodynamic therapy (PDT) to treat OSCC [99,100].

The mammalian target of the rapamycin (mTOR) signaling pathway plays a key role in regulating metabolic processes in cells [101]. The subunits mTORC1 and mTORC2 are responsible for cell growth and survival/proliferation, respectively [102]. The highly selective rapamycin and its analogs (rapalogs) inhibit mTOR by binding to a separate domain from the catalytic site [103]. Rapamycin used in its original form is inadequate due to poor water solubility, absorption, and bioavailability [85]. Therefore, the three rapalogs, temsirolimus, everolimus, and sirolimus, have been developed to serve similar functions as rapamycin [104,105,106]. Temsirolimus is an intravenous prodrug that is converted into rapamycin after injection [85]. In a study assessing the effect of temsirolimus on OSCC associated with bone destruction, temsirolimus successfully decreased the migrative and proliferative nature of HSC-2 OSCC cells [107].

The COX-2 inhibitor celecoxib selectively blocks COX-2, which is always over-expressed in many tumors, including OSCC [85,108]. COX-2 is known to promote proliferation, anti-apoptosis, angiogenesis, inflammation, invasion, and metastasis in cancer cells. These characteristics highly suggest that administration of COX-2 inhibitors and celecoxib can effectively inhibit adhesion, migration, invasion, and metastasis of cells of the human tongue squamous cell carcinoma [109]. This implies the potential application of celecoxib in OSCC treatment, and studies have indeed shown positive results regarding its efficacy of celecoxib in suppressing cell migration and invasion of OSCC [110,111].

## 4. Immune Checkpoint Inhibitors

The two widely adopted, FDA-approved antibodies pembrolizumab and nivolumab target the programmed cell death protein 1 (PD-1) [86,112]. PD-1 inhibitors are immune checkpoint inhibitors (ICIs), which prevent T cell inactivation in the presence of tumor cells. In the absence of inhibitors, PD-1 of the T cell binds with PD-L1 of the tumor cell, blocking T cells from attacking tumor cells [85]. Pembrolizumab and nivolumab bind to the epitopes of the PD-1 molecule with high affinity and high selectivity, thus inhibiting the inactivation of T cells [113]. The newly developed durvalumab and atezolizumab also bind to PD-L1 of tumor cells [86]. Durvalumab as well as atezolizumab are currently under phase III clinical trials for HNSCC treatment, and atezolizumab has shown promising results [112,114].

The effect of ICIs on OSCC and other malignancies reported by researchers has brought great attention to these drugs [85]. However, despite the promising results claimed by these studies, the overall success rates of nivolumab and pembrolizumab applied to HNSCC are approximately 13–18% [86,115,116]. In addition, the possible toxicities and high cost of ICIs pose limitations with respect to clinical practice. Given these limitations of ICIs, the role of biomarkers has become extremely important. Some potential immune biomarkers of HNSCC include PD-L1 expression on tumor cells, tumor-infiltrating lymphocytes (TILs), tumor mutational burden (TMB), and microbiota [86,117].

Although PD-L1^+^ tumors tend to exhibit a higher response to PD-1/PD-L1 inhibitors than PD-L1^−^ tumors do, there is some controversy about whether PD-L1 is a highly reliable biomarker [86]. First of all, the experimental results for PD-1/PD-L1 inhibitors are inconsistent [118]. Furthermore, about 60% of patients who receive PD-1/PD-L1 therapy show primary resistance [86,119]. It is important to note that PD-L1 expression might be regulated by multiple signaling pathways, which include various enzymes (i.e., MAPK, PI3K) that are known to be frequently altered in HNSCC [120]. The expression of PD-L1 is also not exclusive to tumor cells as T cells, natural killer cells, and antigen-presenting cells express this protein in high levels as well [121].

TIL is another potential prognostic biomarker for determining ICI response. In regard to anti-PD-1/PD-L1 therapy, the amount and location of TILs within a tumor are strong indicators for ICI outcomes [122,123]. However, these results have only been confirmed in melanoma and non-small cell lung carcinoma (NSCLC) and not in HNSCC [86,124,125,126].

TMB is an emerging biomarker approved for ICIs. It measures the amount of mutations existing in tumors using a whole genome sequencing [127]. This biomarker demonstrated potential as a promising biomarker of ICI efficacy in various tumors, including HNSCC [128,129]. Nonetheless, the lack of uniformity in methods used to measure TMB necessitates the standardization of TMB calculations [86].

Although many of these biomarkers have presented positive results, each holds challenges that need to be overcome, including validation in HNSCC patients. Recently, some researchers have suggested the oral microbiota are a potential immune biomarker for HNSCC [86,130]. In the process of determining the oral microbiota, 16s rRNA high throughput sequencing is used [86]. Altogether, the rise of ICIs and their biomarkers emphasize the importance of continuous research in this field and the adoption of NGS in the processes.

## 5. Differential Expressions of miRNAs

Composed of around 22 nucleotides, microRNAs (miRNAs) are small, non-coding RNA molecules that regulate gene expression in various organisms. miRNAs modulate gene expression by inhibiting mRNA translation, which then initiates cellular processes such as cell proliferation, differentiation, or apoptosis. It is crucial to understand miRNAs in oncology, as many studies have shown that their dysfunction is responsible for regulating apoptosis and cancer formation [131]. Understanding the role of miRNAs in malignancy will also determine their potential as biomarkers or drug targets. The conventional diagnostic method used for head and neck cancers (HNCs) is a clinical examination by a professional and examination of the relevant histopathology (Brinkmann et al. 2011). Several studies have shown that differential expressions of miRNA help to differentiate cancerous tissue from benign tissue. miRNA biomarkers can be used as diagnostic tools without the need for an invasive procedure [132]. Circulating miRNAs are expressed at different levels depending on the stage of cancer. Therefore, miRNAs provide information that helps with OSCC identification, assessment, and decision-making. Although most previous studies have used qRT-PCR and microarray methods to identify miRNAs involved in carcinogenesis, some recent studies have used NGS. NGS is much more advantageous than qRT-PCR for identifying novel miRNAs, because it does not require knowledge of the miRNA sequences in advance [133].

There are oncogenic and suppressive miRNAs that are up-regulated or down-regulated, respectively, in tumor cells. The up-regulation or over-expression of miRNAs promotes tumorigenesis, while the down-regulation of miRNAs suppresses tumorigenesis. The miRNAs that have been identified using the NGS method are listed in Table 3 according to their regulatory function. The most commonly identified miRNA was miR-21, which is known to be up-regulated in various types of cancer such as breast cancer, OSCC, and gastric cancer [134,135,136,137].

Numerous studies have identified miRNAs associated with OSCC using NGS. One of these studies focused on circulating miRNAs that are associated with OSCC recurrence, and sought to identify the dysregulation of plasma miRNAs in OSCC and OSCC recurrence post-surgery [138]. This study showed that the differential expressions of miR-92b-3p, miR-375, and miR-486-5p were associated with the risk of OSCC recurrence 9–12 months postoperatively. Comprehensive analysis of the NGS data and qRT-PCR revealed that miR-92b-3p was expressed significantly more in postoperative samples than it was in preoperative samples. Furthermore, the miR-92b-3p expression level was much higher in the postoperative samples from patients without OSCC recurrence than it was in those with recurrence [138].

Similarly, miR-375 was significantly up-regulated in postoperative samples compared to pre-operative samples [138]. These findings correspond to the NGS data and suggest that miR-375 is up-regulated postoperatively. The expression levels of miR-375 were also slightly higher in healthy samples than they were in pre-operative samples, as suggested by NGS and qPCR data. The miR-375 expression was also significantly elevated in the post-operative samples of patients without OSCC recurrence compared to those with recurrence. However, miR-375 expression showed no variation from pre- to postoperative in patients with OSCC recurrence (using qPCR validation). Therefore, data suggest that miR-375 expression level is associated with OSCC recurrence [138]. A previous study found that miR-375 was down-regulated in the tissue, saliva, and oral rinse samples from OSCC patients [144]. Therefore, miR-375 may be useful in monitoring OSCC recurrence after surgery [144].

MiR-486-5p is the most promising diagnostic biomarker for OSCC among the three primary miRNAs [138]. The expression of miR-486-5p was significantly lower in pre-operative samples than in healthy samples. In addition, an increase in miR-486-5p expression was apparent in post-operative samples. miR-486-5p is likely associated with OSCC recurrence 9–12 months after surgery, because the expression level of miR-486-5p is not highly elevated in post-operative samples compared to those of pre-operative samples in OSCC patients with recurrence. Altogether, these results suggest that miR-486-5p is a useful biomarker for monitoring OSCC recurrence after surgery. Although previous studies have also reported miR-486-5p to be down-regulated in OSCC tissues, they were the first to describe the role of circulating miR-486-5p in OSCC [138]. These results suggest that circulating miR-486-5p can be identified as a tumor-suppressive miRNA and a strong indicator of OSCC recurrence. The other miRNAs, including miR-486-5p and miR-375, are considered moderate risk indicators of OSCC recurrence 9–12 months after surgery [138].

**Figure 2:**
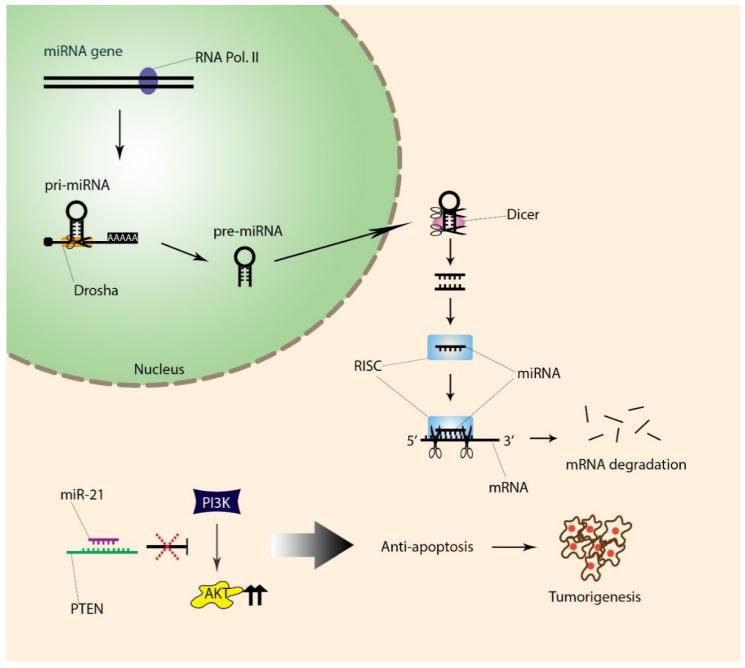
Stages of miRNA formation and tumor growth resulting from reduced expression of *PTEN*. (1) In the nucleus, the miRNA gene is transcribed by RNA Polymerase II (RNA Pol. II), and the resulting product is primary miRNA (pri-miRNA). (2) The pri-miRNA is then modified into precursor miRNA (pre-miRNA) by Drosha. (3) Dicer then trims the pre-miRNA into shorter (21-23bp), double-stranded miRNA in the cytoplasm. (4) One of the strands is fixed to the RNA-induced silencing complex (RISC). (5) When the fixed miRNA strand in the RISC binds to a perfectly complementary mRNA strand, degradation of mRNA occurs, thereby suppressing the expression of target mRNA. (6) The binding of miR-21 to *PTEN* results in reduced expression of *PTEN*. (7) The decrease in *PTEN* activates the PI3K/AKT pathway instead of suppressing it. (8) Constant activation of AKT occurs, leading to cell survival.

Multiple OSCC-related miRNAs and their putative targets were revealed in a study that used Chinese hamsters (*Cricetulus griseus*) as a disease model [141]. This species is ideal as it shares similar buccal tissues with humans. They discovered 11 of the previously identified miRNAs: crg-miR-130b-3p, crg-miR-142-5p, crg-miR-21-3p, crg-miR-21-5p, crg-miR-542-3p, crg-miR-486-3p, crg-miR-499-5p, crg-miR-504, crg-miR-34c-5p, crg-miR-34b-5p, and crg-miR-34c-3p. They also found the following three novel miRNAs: Novel-117, Novel-118, and Novel-135. The expression levels of both miR-21-3p and miR-21-5p were increased in OSCC. As a result, hundreds of related genes were found. The most common gene targeted by miR-21 was *PTEN*. Mutation or deletion of *PTEN* leads to continuous activation of AKT, which increases anti-apoptotic gene expression (Figure 2). In squamous cell carcinoma tissue, the expression level of Bcl-2 was higher when Caspase-3, Caspase-9, and Bax were low. The comprehensive analysis of these results supports the idea that miR-21 may contribute to the progression of OSCC by inhibiting the expression of *PTEN*. *PTEN*, when expressed, inhibits the activation of PI3K to AKT. Therefore, the suppression of *PTEN* causes continuous AKT activation. As the PI3K/AKT pathway is involved in apoptosis and tumorigenesis, mutation or hindrance in *PTEN* is likely to lead to cancer [141].

miR-21 not only serves as a promising biomarker, but also as a potential therapeutic target. *PTEN* is widely known as a tumor-suppressive gene, and the frequency of *PTEN* mutation in various types of human cancers is comparable to that of p53 [145]. Given that *PTEN* plays a key role in human hepatocellular cancer (HCC), one study used luciferase reporter to confirm the relationship between miR-21 and *PTEN* [146]. This group found that miR-21 directly binds to the 3′-UTR of *PTEN* and regulates gene expression [146]. Other studies have also suggested that *PTEN* plays a role in metastasis [145,147].

In one study, miR-21-3p was significantly up-regulated in OSCC tissues [138]. To determine the function of miR-21-3p, TW1.5 cells were treated with miR-21-3p antagomirs (inhibitors). They found that inhibiting miR-21-3p suppressed cell colonization, but had a negligible effect on cell proliferation. In addition, inhibiting miR-21-3p expression in TW1.5 cells suppressed the migration and invasive characteristics of cancer cells. These results together suggest that overexpression of miR-21-3p in OSCC tissues and the dysfunction of this miRNA is associated with OSCC metastasis. This was the first study to reveal that miR-21-3p is highly expressed in OSCC tissues compared to adjacent normal tissues, and that its expression levels are associated with its invasive ability [138].

The oncogenic characteristic of miR-21 serves another advantage to OSCC patients. Oral brushing specimens from OSCC patients revealed that a set of miRNAs were detected, and three of them were overexpressed (miR-21, miR-191, and miR-146) [148]. This suggests that miR-21 has potential use as a non-invasive biomarker.

Plasma samples from healthy individuals, oral leukoplakia (OL) patients, and OSCC patients were collected for analysis [142]. Among the miRNAs identified, three miRNAs (miR-150-5p, miR-222-3p, and miR-423-5p) were differentially expressed across the three groups. miR-222-3p was notably down-regulated in OL patients compared to the normal and OSCC patient groups. However, miR-150-5p and miR-423-5p were significantly up-regulated in OSCC patients relative to normal and OL patient groups. These results indicate the possibilities of the three miRNAs serving as putative biomarkers for diagnosing OL and OSCC. Furthermore, miR-222-3p and miR-423-5p were down-regulated when tumors metastasized to the lymph node. Their expression levels gradually declined with tumor progression in OSCC patients. Interestingly, miR-150-5p expression levels did not correlate with lymph node metastasis and tumor progression but were lower at the later stage of tumorigenesis. As a result, this group suggested that miR-222 and miR-423-5p are useful indicators of tumor progression [142].

In efforts to provide OSCC patients with non-invasive biomarkers for early detection, miRNA expression profiles (miRNome) of OSCC and normal oral mucosa (NOM) were determined [140]. They identified and validated novel diagnostic miRNAs and combinations of miRNAs in plasma samples and formalin-fixed paraffin-embedded (FFPE) tissue samples. The group used NGS to define the complete miRNA expression profiles (miRNome) in OSCC samples to identify the most clinically significant deregulated miRNAs. In normal oral mucosa, 512 mature miRNAs were expressed. Among the 512 miRNAs, three significantly expressed miRNAs (miR-21-5p, miR-143-3p, and miR-148a-3p) accounted for 89% of all reads. In addition, 567 mature miRNAs were detected in the OSCC samples and the three most expressed miRNAs were miR-143-3p, miR-22-3p, and miR-21-5p. While microarray or qRT-PCR only focuses on individual miRNA alterations, the NGS approach allows for a more substantial analysis of the miRNome. By adopting this in-depth approach, this group discovered that only a few miRNAs were significantly expressed in OSCC and that these accounted for most of the miRNome. It is also notable that most of the highly expressed miRNAs were not altered between NOM and OSCC [140].

The discovery of miRNAs that are involved in OSCC tumorigenesis and the characterization of their roles in this setting are important in the therapeutic management of OSCC. Past studies have successfully targeted miR-21, which is the most frequently observed miRNA in OSCC. Inhibiting miR-21 function with antisense miR-21 oligonucleotide has effectively suppressed tumorigenesis and induced apoptosis in TSCC [149]. In addition, the use of peptide nucleic acids (PNAs)-antimiR-21 in breast cancer has shown therapeutic potential [150].

## 6. Conclusions

We have discussed the frequently identified gene alterations and miRNAs that are associated with the development and progression of OSCC. Although many studies have confirmed various gene mutations and miRNAs related to OSCC, more research is necessary for a deeper understanding of the molecular processes involved in tumorigenesis. In addition, NGS plays a crucial role in novel discoveries, but its clinical capabilities are not yet being fully applied. Numerous studies have used qRT-PCR or microarray to confirm the existence of known miRNAs. However, only a few studies have discovered novel miRNAs using NGS despite the introduction of NGS over a decade ago. To highlight the importance of NGS application in OSCC studies, this paper not only discusses the use of NGS in identifying a malignancy, but also implies the need for further research using this technique.

Interesting research questions can be derived from studies discussed in this review. Several papers have mentioned the possibility of particular gene alterations appearing in higher proportions in certain ethnicities. It will be important to identify any ethnicity-associated mutations to optimize OSCC prevention and treatment. A previous study has also suggested the increasing prevalence of OSCC in younger patients. These reasons emphasize the need for more research regarding OSCC-related miRNAs using a high-throughput method for accurate and efficient sequencing.

## Figures and Tables

**Table 1:** List of common gene mutations pertaining to OSCC and their roles in tumorigenesis. Reference sequences and representative driver mutations of each gene are also listed.

Genes/Driver Mutations	Research Method	Type of Variant	Location of Mutation	Regulatory Function	References
**TP53** NM_000546.5 (p.Arg248Trp) (p.Arg213Gln) (p.Pro72Arg) (p.Pro152Leu) (p.Val274Gly) (p.Val274Phe) (p.Glu204Ter*) (p.Gln165Ter*)	Ion Torrent [48,53,54,56,58,61,62,63] Ion AmpliSeq [55] Ion Proton [60] Illumina [57,59,62]	Missense [48,53,54,55,56,57,58,59,60,61,63] Nonsense [48,53,54,56,57,58,59,60,63] Deletion [54,55,56,57,58,60] Insertion [55,56,57,60] Splice-site mutation [53,54,56,59,60,63]	DNA-binding domain [53,54,56]	p53 induces apoptosis (tumor suppressor)	[48,53,54,55,56,57,58,59,60,61,62,63]
**CDKN2A** NM_000077 (p.Pro81His) (p.Arg80Ter*) (p.Trp110Ter*)	Ion Torrent [53,54,56,58,61,63] Ion Proton [60] Illumina [59]	Missense [54,56,59,60] Nonsense [54,56,58,60,63] Deletion [54,58,60,63] Insertion [54,56,60,63] Splice-site mutation [54,56,60,63]	Ankyrin repeats [56]	p16 and p14 function as tumor suppressors	[53,54,56,58,59,60,61,63]
**NOTCH1** NM_017617.5 (p.Gly310Arg) (p.Asp352Gly) (p.Arg365Cys) (p.Thr1014Met) (p.Cys1383Tyr) (p.Gln1957Pro)	Ion Torrent[48,54,56] Ion Proton[60] Illumina[49,59]	Missense [48,54,56,59,60] Nonsense [48,60] Insertions [54,56] Deletions [56] Splice-site [54,56]	EGF-like repeats [48,49,54,56]	Cell growth and division	[48,49,54,56,58,59,60]
**HRAS** NM_005343 (p.Gly12Ser) (p.Gly13Ser) (p.Gly13Arg) (p.Gly13Val)	Ion Torrent [53,58,61,63] Ion AmpliSeq [55] Ion Proton [60] Illumina [59]	Missense [55,58,60,63]		Cell growth and division	[53,55,58,59,60,61,63]
**PIK3CA** NM_006213.2 NM_006218.1 (p.Glu545Lys) (p.His1048Ser) (p.His1047Arg) (p.Glu542Ala) (p.Glu542Lys) (p.His1047Arg)	Ion Torrent [53,54,58,63] Ion AmpliSeq [55] Ion Proton [60] Illumina [57,59]	Missense [55,56,57,58,59,60,63] Nonsense [63] Splice-site [60]		Cell growth and division	[53,54,55,57,58,59,60,63]
**CASP8** NM_001080125 (p.Ile354Asn) (p.Arg472Ter) (p.Cys404Tyr)	Ion Torrent [56,62]	Nonsense [56] Deletion [56]	Caspase homologues domain [56]	Regulates cell apoptosis	[56,62]
**FBXW7** NM_033632.3 (p.Ser462Phe)	Illumina [57] Ion Torrent [54,61] Ion Proton [60]	Missense [54,57,60]		Tumor suppressor	[57,60,61]
**RB1** NM_000321.3 (p.Ile680Thr)	Ion Torrent [58,61] Ion Proton [60]	Missense [60] Nonsense [60]		Tumor suppressor	[58,60,61]
**PTEN** NM_000314.4 (p.Arg161Lys) (p.His185Tyr) (p.Val249Met)	Ion AmpliSeq [55] Ion Proton [60]	Missense [55] Nonsense [60] Deletion [55]		Cell growth and division	[55,60,63]
**EGFR** NM_005228 (p.Ile107Val)	Ion Torrent [54,56,58,59,63] Ion AmpliSeq [55] Illumina [57]	Missense [56] Insertion [59] Deletion [58]	Furin-like repeats [56]	Regulates cell proliferation	[53,54,55,56,57,58,59,63]

**Table 2:** Representative drugs currently available or under investigation for the treatment of OSCC [85,86,87]. Only cetuximab, pembrolizumab, and nivolumab have been approved by the FDA for application on HNSCC/OSCC treatment.

p53 Targeted	*EGFR* Targeted	VEGF Targeted	mTOR Inhibitors	PD-1 Targeted	Others
PRIMA-1	Cetuximab	Bevacizumab	Rapamycin	Pembrolizumab	COX-2 inhibitor
PRIMA-1^MET^ (APR-246)	Nimotuzumab	Aflibercept	Temsirolimus	Nivolumab	-
MIRA-1	Gefitinib	Sorafenib	Everolimus	Durvalumab	-
STIMA-1	Erlotinib	Vandetanib	Sirolimus	Atezolizumab	-
COTI-2	-	-	-	-	-

**Table 3:** miRNAs discovered using NGS and their regulatory functions. Illumina was used in all studies listed below.

Oncogenic (Up-Regulated) miRNA	Suppressive (Down-Regulated) miRNA
miR-21: [138,139,140,141]	miR-92b: [139]
miR-22: [140]	miR-199: [143]
miR-26a: [139]	miR-214: [143]
miR-34c: [141]	miR-375: [139]
miR-34c: [141]	miR-486: [139]
miR-34b: [141]	miR-504: [141]
miR-117: [141]	miR-499: [141]
miR-118: [141]	miR-486: [141]
miR-130b: [141,142]	
miR-135: [141]	
miR-142: [141]	
miR-143: [140]	
miR-148a: [139]	
miR-150: [142]	
miR-221: [142]	
miR-222: [142]	
miR-423: [142]	
miR-542: [141]	
miR-1269a: [143]	
